# Methimazole associated eosinophilic pleural effusion: a case report

**DOI:** 10.1186/s40360-017-0121-1

**Published:** 2017-03-21

**Authors:** Pedro Gaspar-da-Costa, Filipa Duarte Silva, Júlia Henriques, Sónia do Vale, Sandra Braz, João Meneses Santos, Rui M.M. Victorino

**Affiliations:** 1 Serviço de Medicina 2, Hospital de Santa Maria - Centro Hospitalar Lisboa Norte, Avenida Prof. Egas Moniz, 1649-035 Lisbon, Portugal; 2 Serviço de Endocrinologia, Hospital de Santa Maria - Centro Hospitalar Lisboa Norte, Avenida Prof. Egas Moniz, 1649-035 Lisbon, Portugal; 3 Faculdade de Medicina da Universidade de Lisboa, Lisbon, Portugal

**Keywords:** Eosinophilic pleural effusion, Thionamides, Methimazole, Hypersensitivity reaction, Case report

## Abstract

**Background:**

Adverse reactions associated to anti-thyroid drugs include fever, rash, arthralgia, agranulocytosis and hepatitis that are thought to be hypersensitivity reactions. Five cases of pleural effusion associated to thionamides have also been reported, two with propylthiouracil and three with carbimazole.

**Case presentation:**

We report here a case of a 75-year-old man admitted because of unilateral pleural effusion. The patient had a recent diagnosis of hyperthyroidism and 6 days after starting methimazole complained of pleuritic chest pain. He had elevated C-reactive protein and erythrocyte sedimentation rate and normal white blood cell count and liver enzymes. Chest radiography showed a moderate right pleural effusion and the ultrasound revealed a loculated effusion that was shown to be an eosinophilic exudate.

**Conclusions:**

The temporal relationship between methimazole intake and the development of pleural effusion combined with the extensive exclusion of alternative causes, namely infectious, neoplastic and primary auto-immune diseases, led to the diagnosis of hypersensitivity reaction to methimazole. The thionamide was stopped and corticosteroid was started with complete resolution of the pleural effusion in 3 months.

Awareness of this rare adverse reaction of anti-thyroid drugs is important and methimazole can be added to the list of possible etiologies of drug-induced eosinophilic pleural effusion.

## Background

Methimazole or thiamazole, together with carbimazole and propylthiouracil, are anti-thyroid medications belonging to the class of thionamides [1]. Methimazole is the active metabolite of carbimazole and has similar pharmacological activity. Thionamides side effects are rare and include fever, rash, arthralgias, agranulocytosis and hepatitis [1]. Pleuro-pulmonary complications of these drugs are even rarer.

The mechanisms of thionamides side effects are poorly understood but are generally considered to be dose independent by immunologic hypersensitivity. Antineutrophil cytoplasmic antibody (ANCA)-positive vasculitis associated to anti-thyroid drugs have been well described [2–12]. Additionally, a drug-induced lupus-like syndrome and hypersensitivity reactions with cutaneous, renal or pulmonary involvement have also been identified [13–17]. Recently, five cases of pleural effusion induced by anti-thyroid drugs, two with propylthiouracil and three with carbimazole, have been reported in the literature [18–22].

We describe here a patient with hyperthyroidism who developed an unilateral eosinophilic pleural effusion a few days after the initiation of methimazole therapy. An extensive diagnostic evaluation excluded infectious and neoplastic diseases as well as primary auto-immune or vasculitic disease as the cause of serositis. Pleural effusion was thus considered as a manifestation of methimazole-hypersensitivity reaction. To the best of our knowledge, this is the first case of methimazole-induced pleural effusion described in the literature.

## Case presentation

A 75-year-old man was admitted with unilateral right pleural effusion. He presented with pleuritic chest pain that started 4 days before and had no other relevant complaints, namely fever, diaphoresis, dyspnoea, cough, haemoptysis, rash or arthralgia.

He had a past medical history of arterial hypertension, atrial fibrillation, end-stage renal disease on haemodialysis, peripheral artery disease and prostatic hyperplasia. His ambulatory prescription consisted of enalapril, carvedilol, nifedipine, aspirin, warfarin, omeprazole and tansulosin, for more than 3 years. Amiodarone had been suspended, 2 months before, due to ocular toxicity. There was no evidence of thoracic trauma or previous asbestos exposure.

Ten days before hospital admission the patient had an episode of unstable angina. He underwent coronary angiography which showed a lesion in the circumflex artery and angioplasty and implantation of an intracoronary stent coated drug were performed. He started treatment with clopidogrel. By this time blood tests showed low TSH (0.055 uU/mL; reference range: 0.55–4.78 uU/mL) and high FT4 (1.81 ng/dL; reference range: 0.80–1.76 ng/dL) and FT3 (4.84 pg/mL; reference range: 2.3–4.2 pg/mL). Methimazole was started. There was no evidence of pleural effusion at that time.

At hospital admission the patient was afebrile, with a respiratory rate of 18 cycles per minute and oxygen saturation of 95% on room air. His blood pressure was 131/63 mmHg and heart rate was 76 beats per minute. Cardio-pulmonary examination revealed arrhythmic heart sounds, diminished breath sounds on the lower half of the right lung and dullness on percussion at that site was noted. All other findings on physical examination were unremarkable.

Laboratory tests showed normal leucocytes (6 510/uL) and eosinophils (320/uL) counts, elevated C-reactive protein (10.9 mg/dL) and erythrocyte sedimentation rate (58 mm) and normal liver enzymes; TSH (0.019 uU/mL) was low and FT4 (2.51 ng/dL) and FT3 (7.22 pg/mL) were high; INR (1.13) was not in therapeutic range. Arterial blood gases, while the patient was in room air and after haemodialysis, showed hypoxemia (PO_2_: 57.6 mmHg) and metabolic alkalosis (pH: 7.48, PCO_2_: 37.8 mmHg, HCO_3_^−^: 28.9 mmol/L). Electrocardiogram revealed atrial fibrillation rhythm without acute ischaemic changes. Chest radiography showed a homogenous opacity in the lower half of the right pulmonary field and chest ultrasound findings were suggestive of pleural effusion with septa. Right-sided diagnostic thoracentesis was performed. The aspirated pleural fluid had 2277 cells/uL with 70% being macrophages, 18% eosinophils and 12% neutrophils. The effusion protein/serum protein ratio was 0.7 and the effusion lactate dehydrogenase (LDH)/serum LDH ratio was 1.8. Pleural fluid cultures revealed no bacteria or mycobacteria and no malignant cells were detected on cytological examination of pleural fluid. Bronchofibroscopy did not reveal any macroscopic lesion. Bronchoalveolar fluid cultures were negative and the cytological analysis of the fluid showed no malignant cells. Pleural biopsy was not performed due to high bleeding risk as the patient was on double anti-platelet and anticoagulation therapy. The blood cultures yielded no bacteria or mycobacteria and blood interferon gamma release assay for *Mycobacterium tuberculosis* was negative. Anti-nuclear antibodies (ANA), anti-double stranded DNA (anti-dsDNA) antibodies and ANCA were negative.

Thoracic, abdominal and pelvic computed tomography (CT) was performed and confirmed the moderate right pleural effusion with no other positive findings. Thoracic CT angiography excluded pulmonary embolism. Although Dressler syndrome was considered [23], it usually occurs after myocardial infarction rather than unstable angina and moreover, the short interval between the cardiac event and the pleural effusion did not support this hypothesis [24].

Titers of thyroglobulin (Tg), anti-Tg, anti-thyroperoxidase and anti-TSH receptor antibodies were normal. The cervical ultrasonography revealed thyroid micronodules and there was no radioisotope uptake in the 99mTechnetium thyroid scintigraphy. Hyperthyroidism was interpreted as type II amiodarone induced thyrotoxicosis.

After the exclusion of neoplastic, infectious and primary auto-immune diseases, considering an exudative pleural effusion with significant eosinophilia and a well-established temporal relation with the initiation of methimazole treatment, a diagnosis of methimazole-induced pleural effusion was made. A single organ drug hypersensitivity reaction was assumed in the absence of evidence of other organ involvement. Anti-thyroid medication was suspended, systemic corticosteroid was initiated and all other medications were maintained. Progressive and rapid clinical and laboratorial improvement was evident with normalization of inflammatory biomarkers and thyroid function and resolution of hypoxemia. Three months after methimazole was suspended there was complete resolution of pleural effusion.

## Discussion

Thionamides-induced auto-immune phenomena, including pleuro-pulmonary complications, exhibit usually the features of ANCA-positive systemic vasculitis. Stankus et al. published, in 1992, the first case of propylthiouracil-induced hypersensitivity vasculitis and since then 61 cases of vasculitis associated to anti-thyroid drugs have been reported, most involving propylthiouracil [15, 25]. There are only nine reported cases of carbimazole-induced vasculitis and seven cases of vasculitis associated to methimazole [2–9, 13, 14, 17, 25–29]. In addition, a lupus-like syndrome can be induced by any of the thionamides [8, 13, 14, 21, 30]. Clinical manifestations are similar to systemic lupus erythematosus (SLE) and usually patients have positive anti-nuclear, anti-dsDNA and anti-histone antibodies [21].

Pleuro-pulmonary complications of thionamides are very rare and include alveolar hemorrhage, interstitial pneumonia, pulmonary cavitations and pleural effusion that are considered adverse auto-immune reactions triggered by these drugs [6, 7, 9, 18–22, 31, 32]. Five cases of anti-thyroid drug-induced pleural effusion, two with propylthiouracil and three with carbimazole, have been described in the literature [18–22]. The case reported here represents the first case of methimazole-associated pleural effusion. The temporal relationship between initiation of the drug and the appearance of pleural effusion, the resolution of the effusion after withdrawal of methimazole with administration of corticosteroid, in the absence of other potential cause for pleuritis, is consistent with the diagnosis of drug-induced pleural effusion. Our case differs from the previous reports by the short time between the initiation of the thionamide and the onset of pleural effusion that was only 6 days.

Pleural fluid of the two patients with propylthiouracil associated pleuritis was an eosinophilic exsudate, as in the case reported here [18, 19]. Pleural biopsy was made in one of these patients and showed chronic inflammation of the pleura with prominent eosinophils and both patients had negative auto-immune markers. None of the three patients with carbimazole-induced pleuritis had an eosinophilic exudate [20–22]. Two of these three reported cases had pleural nonspecific inflammatory infiltrates and negative auto-immunity, namely ANA and ANCA. The third patient had positive ANA, anti-dsDNA and anti-histone antibodies and pleuritis was considered as a drug-induced SLE-like syndrome.

As stated before, the mechanisms involved in these cases are most likely non-dose dependent, exactly like in drug induced liver injury where lesions occur via immunologic hypersensitivity [33] by generation of toxic metabolites [34] or alternatively metabolites that induce an immunological reaction [34, 35]. The inflammatory response with abundant eosinophils in the case reported here suggests that this represents a T cell drug hypersensitivity reaction with polarization to T helper 2 cells producing interleukin 4 and interleukin 5 that are known to induce eosinophil differentiation. Interestingly, a recent report by Uematsu et al. [36], identified a T helper 2 biased lymphocyte response in a model of methimazole acute liver injury mediated by microRNAs [36]. Another area of increasing interest is that of risk factors for methimazole adverse reactions. Thus, HLA-B*27:05 and other single nucleotide polymorphisms were identified as possible risk factors for anti-thyroid agranulocytosis [37], and the prospect that certain microRNAs with effects on lung development might represent prognostic tools in thyroid-dependent lung disease [38] has also been recently discussed.

The clinical and radiological evolution of this case was identical to the cases of pleural effusion induced by other thionamides. The anti-thyroid drug considered to be associated to the effusion was withdrawn in all patients and alternative anti-thyroid therapy options were initiated in three of the five patients [19, 20, 22]. The cessation of the drug was followed by resolution of the effusion within 3 to 5 months. Two of the five patients were given corticoesteroid for a short period of time and in decreasing dosages and in these cases a more rapid resolution of serositis was observed [19, 21]. In the present case, a diagnosis of type II hyperthyroidism, due to cytotoxic effect of amiodarone on thyroid cells, was made and thus maintenance of thionamides treatment was not required.

## Conclusions

In conclusion, we describe here the first case of eosinophilic pleuritis associated to methimazole due to an hypersensitivity immune drug reaction with full remission after withdrawal the drug. Awareness of clinicians about the possibility of this drug adverse reaction may help identification of future similar cases.

## Abbreviations

ANA: Anti-nuclear antibodies
ANCA: Antineutrophil cytoplasmic antibody
Anti-dsDNA: Anti-double stranded DNA antibodies
CT: Computed tomography
SLE: Systemic lupus erythematosus
Tg: Thyroglobulin
